# User-Reported Comfort, Functionality, and Satisfaction Among Pediatric Lower Limb Amputees Using Conventional Prosthetic Devices: A Multi-Center Cross-Sectional Study

**DOI:** 10.3390/children13081013

**Published:** 2026-07-30

**Authors:** Siphosethu Mgwili-Qikani, Guillermo Alfredo Pulido Estrada, Constance Rufaro Sewani-Rusike

**Affiliations:** 1 Department of Rehabilitation Medicine, Faculty of Medicine & Health Sciences, Walter Sisulu University, Mthatha 5100, South Africa; 2 School of Public Health, Faculty of Medicine & Health Sciences, Walter Sisulu University, Mthatha 5100, South Africa; gpulidoestrada@wsu.ac.za; 3 School of Human Biology, Faculty of Medicine & Health Sciences, Walter Sisulu University, Mthatha 5100, South Africa

**Keywords:** pediatric amputation, prosthetic outcomes, patient-reported outcomes, rehabilitation, satisfaction, lower limb prosthetics

## Abstract

**Highlights:**

**What are the main findings?**

• Comfort and functionality were strongly associated with overall satisfaction among pediatric lower-limb prosthesis users.
• Children with congenital limb loss reported higher satisfaction with their prosthetic devices than those with acquired amputations.

**What are the implications of the main findings?**

• Optimizing prosthetic comfort and functional performance may improve long-term prosthesis acceptance and user satisfaction in children.
• Patient-centered, growth-responsive rehabilitation strategies should be prioritized to support positive prosthetic outcomes in pediatric populations.

**Abstract:**

**Background/Objectives:** To determine user-reported comfort, function, and overall satisfaction among pediatric lower limb amputees with conventional prosthetic devices and to identify possible factors associated with overall satisfaction. **Methods:** A cross-sectional observational study was conducted at three tertiary orthotic and prosthetic centers in the Eastern Cape, South Africa. Caregiver–child dyads completed a structured questionnaire evaluating perceived prosthetic comfort, functionality, and satisfaction. There were fifty-two unilateral pediatric lower limb amputees aged 4–17 years who had used conventional prosthetic appliances for at least one year. Questionnaire responses were summarized using descriptive statistics. Spearman’s rank correlation and non-parametric tests were used to examine associations among study variables. Multiple linear regression analysis was performed to identify factors independently associated with overall satisfaction. **Results:** Mean comfort was 3.54 (SD = 0.87), functionality satisfaction was 3.42 (SD = 0.78), and overall satisfaction was 2.73 (SD = 0.79). Comfort had strong correlations with functionality (ρ = 0.63, *p* < 0.001) and overall satisfaction (ρ = 0.69, *p* < 0.001). The satisfaction difference between congenital and acquired amputations was statistically significant (*p* = 0.006), with those with congenital amputation reporting substantially greater satisfaction than those with acquired amputation. Multiple linear regression analysis showed that perceived prosthetic functionality (β = 0.383, *p* < 0.001) and comfort (β = −0.535, *p* < 0.001) were independently associated with overall satisfaction. **Conclusions:** Perceived comfort and prosthetic functionality were independently associated with overall satisfaction among pediatric users of conventional prosthetic devices. These findings support patient-centered prosthetic design and rehabilitation strategies that prioritize socket comfort and functional performance to improve children’s overall satisfaction with their prosthetic devices.

## 1. Introduction

Pediatric lower limb amputation presents unique rehabilitation challenges due to ongoing growth and musculoskeletal development, which can alter limb length, socket fit, and prosthetic alignment [1]. These changes affect gait mechanics and pressures at the limb–socket interface, impacting comfort and functional performance in daily life [2]. Early prosthetic intervention, coordinated by a multidisciplinary team, is essential to address these issues through careful alignment, limb length accommodation, and appropriate component selection [3].

Due to economic and technological limitations, traditional prosthetic systems are still the primary option for pediatric amputees in many low- and middle-income countries. Such systems typically do not have sensors that adapt to take into account rapid developmental changes, necessitating regular clinical recalibration or replacement to sustain functional performance [4]. Although it is common for service delivery models to regard the number of prosthetic follow-up visits as a descriptor of quality, there is increasing evidence in rehabilitation literature that patient-reported outcomes—namely comfort, functional integration, and participation—are better independent correlates of long-term acceptance and use [5]. According to Biddiss and Chau [6], dissatisfaction with a prosthesis is more closely related to socket discomfort and perceived functional limitations than the number of attended clinical consultations. When left unaddressed, poorly fitting and functioning prostheses can lead to further complications such as gait deviations, musculoskeletal pain, or eventual abandonment of the prosthesis [2].

Despite the clinical importance of patient-reported outcomes in prosthetic rehabilitation, there is limited published evidence on the experiences and satisfaction of pediatric prosthetic users in resource-constrained public health systems [4]. Insights into children’s perceptions of prosthetic comfort, functionality, and overall satisfaction are important to the future development of pediatric-specific prosthetic design, rehabilitation strategies, and long-term use of a prosthetic. We therefore assessed user-reported comfort, functionality, and overall satisfaction of pediatric lower limb amputees with conventional prosthetic devices in a multi-center cohort in the Eastern Cape, South Africa, and related this cross-sectional analytical approach to identify factors associated with overall satisfaction. By emulating those parameters, the research fills an essential gap in clinical settings, as there are no validated instruments available that directly cater to elucidating children’s distinct user needs and interaction quality with prosthetics during adaptation [7]. This study aimed to assess user-reported comfort, functionality, and overall satisfaction among pediatric lower limb amputees using conventional prosthetic devices and to identify factors associated with satisfaction.

## 2. Materials and Methods

This cross-sectional observational study used a structured patient-reported outcome questionnaire to evaluate user-reported comfort, functionality, and overall satisfaction in pediatric lower limb amputees using conventional prostheses. The study was conducted across the three public-sector hospitals providing tertiary Orthotics and Prosthetics (O&P) services in the Eastern Cape province of South Africa:

• Nelson Mandela Academic Hospital (Mthatha).
• Frere Hospital (East London).
• Provincial Hospital (Gqeberha).

These facilities served as the primary referral centers for pediatric prosthetic rehabilitation in the province, collectively managing the majority of children requiring prosthetic limb services within the public health sector. Participants receiving prosthetic care at these centers were eligible for inclusion in the study. The study population consisted of pediatric lower-limb amputees aged 4–17 years who had received conventional prosthetic services at one of the participating centers during the available record period for each institution, spanning January 2021 to July 2025. This age range was selected because children in this developmental stage are generally able to provide meaningful, age-appropriate responses regarding comfort, prosthetic functionality, and overall satisfaction, with caregiver assistance provided as needed.

Given the relatively low incidence of pediatric limb loss and the limited number of specialized prosthetic centers within the province, a census sampling approach was adopted. All eligible pediatric prosthetic users managed within this timeframe were identified from institutional records and considered for inclusion.

A total of 75 clinical records were identified across the three centers and screened for eligibility. Following application of the inclusion and exclusion criteria, 20 records were excluded, comprising incomplete clinical records (*n* = 10), bilateral lower-limb amputations (*n* = 5), upper-limb amputations (*n* = 2), and cases without documented prosthetic adjustments during the study period (*n* = 3). No participants were excluded because of the use of specialized prosthetic devices, as there were none. This screening process yielded 55 eligible participants.

Eligible participants were subsequently contacted between June and September 2025, either during routine follow-up visits or telephonically, to complete the patient-reported outcome questionnaire. Of the 55 eligible participants, 52 (94.5%) completed the questionnaire, while three participants either declined participation or could not be reached after repeated contact attempts. The final study sample therefore consisted of 52 pediatric lower-limb amputees, including 27 participants from Frere Hospital, 18 from Provincial Hospital, and 7 from Nelson Mandela Academic Hospital. The larger contribution from Frere Hospital reflects the availability of complete electronic records throughout the study period, whereas the smaller numbers from Nelson Mandela Academic Hospital and Provincial Hospital primarily reflect differences in the duration and completeness of available clinical records across participating centers.

### 2.1. Inclusion Criteria

Participants were eligible for inclusion if they met the following criteria:

• Age between 4 and 17 years.
• Unilateral lower limb amputation.
• Current use of a conventional prosthetic device for at least a year.
• Receiving prosthetic follow-up care at one of the participating hospitals.
• Ability of the child and caregiver to provide responses to the questionnaire.

### 2.2. Exclusion Criteria

Participants were excluded if they met any of the following conditions:

• Bilateral lower limb amputations.
• Use of specialized high-performance prosthetic devices (e.g., sports prostheses).
• Congenital limb deficiencies managed without prosthetic fitting.
• Inability of the caregiver or child to provide questionnaire responses.

Data were collected using a self-developed structured questionnaire. Questionnaire development was informed by the literature and established patient-reported outcome measures, including the General Comfort Questionnaire (GCQ) and the Client Satisfaction Questionnaire (CSQ-8). The questionnaire comprised three single-item domains evaluating comfort, functionality, and overall satisfaction. As each construct was measured by a single global item, assessing internal consistency reliability with Cronbach’s alpha was not appropriate, as the statistic requires multiple items measuring the same underlying construct. Instead, the instrument was pilot tested among caregiver–child dyads to refine wording, improve clarity, and ensure content relevance before data collection.

Responses were recorded using a five-point Likert scale, with higher scores indicating more positive user experiences. Data were entered and analyzed using IBM SPSS Statistics Version 29. Descriptive statistics were used to summarize participant characteristics and patient-reported outcome scores. Categorical variables were presented as frequencies and percentages, while continuous variables were summarized using means and standard deviations (SD) for normally distributed data and medians with interquartile ranges (IQR) for non-normally distributed data.

Categorical variables were analyzed using Chi-square tests or Fisher’s Exact tests, the latter employed when expected cell frequencies were insufficient. Given the ordinal nature of the outcome measures, Spearman’s rank-order correlation coefficients were utilized to evaluate associations between comfort, prosthetic functionality, and overall satisfaction.

A multiple linear regression analysis was conducted to assess the independent associations of perceived comfort and prosthetic functionality with overall satisfaction. Participants with missing data on any regression variables were excluded from the complete-case analysis. Model assumptions were verified by evaluating residual distributions and multicollinearity; the latter was assessed through tolerance values and variance inflation factors. All statistical tests were two-tailed, and a *p*-value < 0.05 was considered statistically significant.

Ethical clearances were obtained prior to the study’s conduct from the Faculty of Health Sciences Ethics Committee at Walter Sisulu University and the Department of Health, Eastern Cape. Furthermore, permission to conduct the study was also granted by the following hospitals: Frere Hospital, Livingston Hospital, and Nelson Mandela Hospital. Informed consent was obtained from parents or guardians, and assent was obtained from children before administering questionnaires.

Grammarly for educational institutional license (https://app.grammarly.com) (24 August 2024) was used for language editing, including grammar, spelling, punctuation, and clarity improvements. The authors reviewed all content and take full responsibility for the final manuscript.

## 3. Results

### 3.1. Demographic Characteristics

The sociodemographic characteristics of the study participants are presented in Table 1. Of the 52 pediatric lower-limb amputees included in the study, the largest proportion was in the 13–17 years age group (42.3%), followed closely by those aged 6–12 years (40.4%). The youngest group, 4–5 years, accounted for 17.3% of the sample. Regarding gender distribution, the majority of participants were male (67.3%), while female participants comprised 32.7% of the study population.

The distribution of the primary reason for amputation according to gender is presented in Table 2. Among the 52 pediatric amputees, the most common reason for amputation was congenital causes (51.9%), followed by trauma (28.8%) and disease-related causes (19.2%). When broken down by gender, 64.7% of females had congenital amputations compared to 45.7% of males. Trauma-related amputations were somewhat more common in males (31.4%) than in females (23.5%), while amputations due to disease were also higher in males (22.9%) compared to females (11.8%). Although congenital amputations appeared proportionally more common among females and trauma- and disease-related amputations appeared proportionally more common among males, these differences were not statistically significant (χ^2^, *p* = 0.412).

The distribution of the primary reason for amputation across age groups is presented in Table 3. Among the 52 pediatric amputees, the distribution of reasons for amputation varied across age groups. In the youngest group (4–5 years), amputations were overwhelmingly due to congenital causes (88.9%), with only one case (11.1%) attributed to trauma and none to disease. In the 6–12 years group, congenital amputations still dominated (57.1%), but there was also a notable proportion due to disease (23.8%) and trauma (19.0%). In contrast, among adolescents aged 13–17 years, trauma (45.5%) became the most common cause, followed by congenital (31.8%) and disease (22.7%).

Fisher’s exact test demonstrated a statistically significant association between age group and primary reason for amputation (*p* = 0.044).

### 3.2. Assessing User-Reported Comfort, Functionality, and Satisfaction Among Pediatric Lower Limb Amputees Using Conventional Prosthetics

Descriptive statistics were computed to summarize participants’ responses on comfort, functionality satisfaction, and overall satisfaction with their prosthetic limbs, and are presented in Table 4. Comfort scores ranged from 2 to 5, with a mean of 3.54 (SD = 0.87), indicating that most participants reported moderate to high comfort levels. Functionality satisfaction showed a comparable distribution, with a mean of 3.42 (SD = 0.78) and a range of 2 to 5, suggesting similar perceptions of prosthetic usability and performance in daily activities. Overall satisfaction, however, recorded a mean score of 2.73 (SD = 0.79), with scores ranging from 1 to 4, indicating greater variability in participants’ overall perceptions of their prosthetic devices. The relatively wide range of responses indicates substantial variability in participants’ overall perceptions of their prosthetic devices, with responses centred around the neutral category.

The distribution of comfort ratings for daily prosthetic use varied across the five-point scale and is presented in Table 5. A total of 48.1% of participants (*n* = 25) reported high levels of comfort (ratings of 4 or 5), indicating generally favorable perceptions of prosthetic comfort. In contrast, 26.9% (*n* = 14) reported discomfort (rating of 2), while 25.0% (*n* = 13) indicated moderate comfort (rating of 3). No participants reported their prosthesis as very uncomfortable (rating of 1). Although the majority of participants experienced acceptable levels of comfort, nearly half of the cohort reported only moderate or low comfort, highlighting persistent challenges in achieving optimal socket fit and comfort among pediatric prosthetic users.

The distribution of overall satisfaction ratings is presented in Table 6. Overall satisfaction was predominantly neutral. Almost half of the participants (48.1%) selected the neutral response, while 15.4% reported being satisfied. A total of 36.6% reported some degree of dissatisfaction, comprising 30.8% who were somewhat dissatisfied and 5.8% who were very dissatisfied. No participant reported being very satisfied. These findings are consistent with the overall mean satisfaction score of 2.73 (SD = 0.79); responses were centered around the neutral category, with a slight tendency toward dissatisfaction.

Satisfaction with prosthetic functionality according to the primary reason for amputation is presented in Table 7. There was a statistically significant association between the primary reason for amputation and satisfaction with prosthetic functionality (Fisher’s Exact Test *p* = 0.006). Children with congenital amputations reported higher levels of prosthetic functionality satisfaction (56%) than those with trauma (13% satisfied, none very satisfied)-or disease-related amputations (0% satisfied/very satisfied). By contrast, dissatisfaction was most pronounced in the disease group (70%) and the trauma group (33.3%). Most trauma cases (53%) reported neutral satisfaction.

Spearman’s rank-order correlation was performed to examine the associations among comfort, functionality satisfaction, and overall satisfaction with prosthetic use. All three variables were measured on 5-point Likert-type scales and are presented in Table 8.

• The correlation between comfort and functionality satisfaction was strong and positive (ρ = 0.63, *p* < 0.01), indicating that participants who reported greater comfort also tended to perceive their prostheses as more functionally satisfactory.
• Comfort and overall satisfaction were also strongly correlated (ρ = 0.69, *p* < 0.01), indicating that greater comfort was associated with higher overall satisfaction.
• Functionality satisfaction and overall satisfaction showed a moderate-to-strong positive relationship (ρ = 0.51, *p* < 0.01), suggesting that perceptions of functional performance are moderately aligned with overall user satisfaction.

All observed coefficients were statistically significant at the 0.01 level (two-tailed), suggesting consistent monotonic relationships across user-experience dimensions. Positive correlations indicate that higher comfort and functionality scores are associated with greater overall satisfaction. The strength of the relationships (ρ > 0.50) indicates that these three domains—comfort, functionality, and overall satisfaction—are closely related constructs, where improvement in one dimension is likely to be associated with proportional gains in the others.

A multiple linear regression analysis was performed to examine the association between perceived prosthetic functionality, comfort, and overall satisfaction, presented in Table 9. The regression model was statistically significant (F(2,47) = 36.713, *p* < 0.001), explaining 61.0% of the variance in overall satisfaction (R^2^ = 0.610; adjusted R^2^ = 0.593). Prosthetic functionality was positively associated with overall satisfaction (B = 0.326, β = 0.383, 95% CI: 0.153–0.499, *p* < 0.001). Comfort was also significantly associated with overall satisfaction (B = −0.415, β = −0.535, 95% CI: −0.574 to −0.257, *p* < 0.001). The negative regression coefficient reflects the reverse coding of the comfort variable, whereby lower numerical scores represented greater perceived comfort. Collinearity diagnostics indicated no evidence of problematic multicollinearity (tolerance = 0.811; VIF = 1.232 for both predictors).

Two participants with missing data were excluded from the regression analysis (complete-case analysis).

## 4. Discussion

This study aimed to assess user-reported comfort, function, and overall satisfaction in pediatric lower-limb amputees with conventional prosthetic devices. Results showed that the majority of respondents reported moderate to high levels of comfort and functionality, whereas overall satisfaction scores centered around the neutral category, indicating greater variability in children’s perceptions of their prosthetic devices. These findings suggest that positive perceptions of comfort and functionality do not necessarily translate into high overall satisfaction and that broader aspects of the prosthetic experience may influence children’s overall perceptions.

This study further demonstrated that comfort and functionality were strongly associated with overall satisfaction. Multiple linear regression analysis showed that perceived comfort and prosthetic functionality were independently associated with overall satisfaction and together explained 61.0% of the variation in satisfaction scores. These findings emphasize the importance of optimizing both comfort and functional performance during pediatric prosthetic rehabilitation while recognizing that additional psychosocial and contextual factors may also contribute to overall satisfaction, indicating the imperative to move towards more patient-centered and adaptable prosthetic solutions [8,9].

### 4.1. Comfort as a Cross-Cutting Theme in Experiences of Prosthesis

Comfort emerged as an important component of the prosthetic user experience, as evidenced by an average score of 3.54 ± 0.87 and by over 48% of respondents describing their prostheses as comfortable or very comfortable. This observation supports previous work demonstrating that comfort within a socket, as well as the residual limb’s tolerance to the prosthesis, are among the factors with the greatest impact on prosthesis acceptance and duration of use, in addition to psychological well-being [6,10,11]. However, recurring issues with socket fit—often exacerbated by the rapid skeletal maturation and soft tissue fluctuations inherent to the pediatric population—continue to pose significant barriers to consistent device use [12].

Despite these generally favorable outcomes, approximately 26.9% of participants reported some degree of discomfort, indicating that socket fit, limb alignment, or suspension stability remain challenges. In addition, these challenges are often compounded in pediatric prosthetic users due to growth-associated anatomical and volumetric changes in the residual limb. Therefore, comfort can be understood as a temporal outcome that develops over time rather than a static product of prosthetic delivery [13]. In addition, certain aspects of prosthetic design—particularly usability and ease of control—can influence comfort, as children may continue to have difficulty stabilizing their device during complex motor tasks [14].

Inadequate socket comfort may result in complications such as periprosthetic skin conditions due to pressure, gait deviations, or reduced use of their prosthesis. As a result, the need for routine monitoring, periodic clinical review, and proactive management of the prosthetic interface in pediatric users is critical to maintaining their optimal comfort [15]. Further, the literature has revealed a notable prevalence (60.2%) of chronic pain among prosthetic users throughout their lifetimes, emphasizing the importance of attention to intra-body biomechanics and ergonomics in reducing device abandonment [16].

These findings highlight the need to prioritize comfort throughout rehabilitation, rather than consider it a milestone achieved at initial prosthetic fitting.

### 4.2. Functional Satisfaction and Etiology of Amputation

The functional satisfaction score with the prosthesis had a mean of 3.42 ± 0.78, indicating moderate functional satisfaction. There was a statistically significant association between amputation etiology and functional satisfaction (*p* = 0.006, Fisher’s Exact Test). Children with congenital limb loss reported higher levels of satisfaction than those who experienced trauma- or disease-related amputation. Participants with disease-related amputations reported the highest level of dissatisfaction.

The discrepancy has potentially been linked to amputee recovery difficulties and comorbidities seen in disease-based amputations that may play a role in both prosthesis fitting and functional mobility [17,18]. On the other hand, timely and even permanent prosthesis application in children with congenital limb deficiencies is usually helpful for the establishment of proprioceptive and motor control patterns that are more integrated, which might improve functional outcomes and satisfaction [19]. Moreover, an early adaptation to limb absence can have positive effects on psychological acceptance of the prosthesis and its integration as part of the body schema instead of a compensatory device [20].

These findings are consistent with previous studies suggesting that individuals born with limb loss show more adaptation in terms of motor function and self-image than those who lose limbs later [21,22]. On the other hand, trauma- or disease-related amputations may result in substantial psychological adjustment, including changes in body image and delayed acceptance of a prosthetic limb [23]. These findings underscore the need for tailored rehabilitation approaches that encompass both the physical and psychological aspects of prosthetic adjustment [15].

In the clinical setting, this highlights the need for tailored rehabilitation protocols that include counseling, graduated mobility progression, and peer support aimed at improving functional confidence and prosthetic satisfaction. Similarly, developing adaptive properties for prosthetic technologies to very well accommodate changing functional demands may further contribute to long-term functional confidence and prosthetic satisfaction [24].

### 4.3. Relationship Between Comfort, Functionality, and Overall Satisfaction

Mean scores comparison reflected the overall high level of comfort and functional performance in the patient participant group, indicating a close relationship between them. The strong correlation observed between comfort, functionality, and overall satisfaction affirms this finding, demonstrating consistency with multidimensional models of prosthetic outcomes [21,25].

In terms of patient satisfaction and prosthetic acceptance, Higher perceived comfort and functionality were associated with higher overall satisfaction [26]. From the user’s viewpoint, overall satisfaction appears to be closely associated with how comfortable a prosthesis is and its functional support of daily activities, rather than purely technical or mechanical aspects of the device [18,26].

This highlights the fact that successful prosthetic rehabilitation is ultimately a biomechanical and subjective phenomenon in which comfort and functional utility are jointly associated with satisfaction and long-term prosthesis use [27]. Moreover, previous studies have reported that those who experience increased mobility and functional independence with their prosthetic devices have been found to report greater overall satisfaction and quality of life [28].

Multiple linear regression analysis demonstrated that perceived comfort and prosthetic functionality were independently associated with overall satisfaction, collectively explaining approximately 61% of the variation in satisfaction scores. These findings reinforce the correlation analyses by demonstrating that both constructs remained independently associated with satisfaction when considered simultaneously. Although comfort exhibited the stronger standardized regression coefficient, both variables contributed significantly to the model, highlighting the importance of optimizing both socket comfort and functional performance during pediatric prosthetic rehabilitation [21]. Nevertheless, approximately 39% of the variability in satisfaction remained unexplained, suggesting that additional factors—such as psychological adjustment, family support, expectations, prosthetic aesthetics, rehabilitation intensity, and environmental influences—may also contribute to children’s overall satisfaction and should be explored in future studies [18,28].

### 4.4. Clinical and Psychosocial Implications

The positive association observed between comfort, functionality, and overall satisfaction is clinically relevant and emphasizes that achieving an optimal fit for the prosthetic as well as functional performance of the prosthetic is critical to improve user well-being and enable a successful integration into daily life. Beyond these physical factors, Previous studies suggest that psychological processes, including expectation management and emotional adaptation, may influence long-term satisfaction [18].

These results advocate for a shift toward a user-centered biopsychosocial model in pediatric prosthetic rehabilitation. Although coping and emotional adjustment were not measured in the present study, previous research suggests that they may contribute to overall satisfaction alongside perceived comfort and functionality [21]. This has the potential to create more engagement with prosthetic rehabilitation and facilitate prosthetic solutions that better reflect children’s developmental needs and daily activity preferences [29].

Clinically, these findings support regular assessment of socket pressure distribution, alignment, suspension systems, and patient education to optimize comfort and functional performance throughout childhood. Further, frequent follow-up and the implementation of growth-responsive prosthetic design features may assist in sustaining comfort and functional performance across time. Furthermore, psychological support, including peer mentoring and family counseling, may enhance positive body image, leading to acceptance of the prosthesis [30].

Psychosocial approaches such as family counseling and play-based methods may be especially effective for children with trauma-related amputations by lowering psychological distress and aiding adaptation to prosthesis use [22,23]. Incorporating these psychosocial approaches with biomechanical optimization could lead to improvements in long-term prosthetic use and, more generally, quality of life [16]. Furthermore, there is an increasing emphasis on directing rehabilitation services to meet these holistic needs, which is important if participants are to facilitate improved patient-reported outcomes and uniformity of care within pediatric prosthetic services [31].

Together with the present regression findings, these observations support multidisciplinary rehabilitation strategies that integrate biomechanical optimization with psychosocial support to enhance patient-reported outcomes among pediatric prosthetic users.

### 4.5. Limitations

Several limitations should be taken into account when interpreting the findings of this study.

First, the cross-sectional design makes it impossible to capture any temporal changes in comfort, functional usability, and satisfaction. Because pediatric prosthetic use is inherently dynamic owing to growth and development, longitudinal studies are warranted that capture how user experiences change over time.

Second, the study sample was small (*n* = 52), typical of the low rates of pediatric lower limb amputation and a small number of specialist prosthetic service centers in the province. Although the sample accounts for a large percentage of the available population from within the Eastern Cape public health system, findings may not be generalized beyond other regions or healthcare settings.

Third, the questionnaire was developed specifically for this study, using items informed by established patient-reported outcome measures, and was pilot-tested to improve clarity and content. However, formal psychometric validation of the adapted instrument was not undertaken. Consequently, the measurement properties of the questionnaire should be further evaluated in future studies.

Fourth, the use of caregiver-assisted responses, especially for younger children, may have introduced response bias. The caregiver may rephrase (or report on) the child’s subjective experience, possibly affecting objective ratings like comfort or satisfaction.

Finally, although the multiple linear regression model explained a substantial proportion of the variability in overall satisfaction (61%), approximately 39% of the variance remained unexplained, indicating that additional factors not assessed in this study may also influence children’s prosthetic experiences. Future research should consider incorporating psychosocial, environmental, prosthetic, and rehabilitation-related variables to develop a more comprehensive understanding of the independent correlates of overall satisfaction.

Despite these limitations, the study provides important evidence on pediatric prosthetic outcomes within a resource-constrained public healthcare setting. By combining patient-reported measures with multivariable analysis, the findings identify perceived comfort and prosthetic functionality as key factors independently associated with overall satisfaction and provide evidence to support patient-centered pediatric prosthetic rehabilitation and future research.

### 4.6. Recommendations

These findings justify prioritizing prosthetic comfort and functional performance as central factors associated with satisfaction among pediatric users. From a clinical perspective, routine follow-up should be systematic in evaluating socket fit, functional performance, and identifying early signs of pain or restricted mobility, complemented where feasible by objective measures (for example, pressure mapping and dynamic adjustments). If rehabilitation is indicated, it should focus on functional training to optimize participation in developmentally appropriate activities. A growth-responsive, user-centered, and multidisciplinary model of care that addresses the physical and emotional demands of prosthetic use is described at the service level (e.g., prosthetists, therapists, and psychosocial support strategies such as family counseling and peer support). At the level of health systems, we need to learn how to move in the direction of patient-centered evaluation frameworks: routine and standardized use of patient-reported outcome measures; training for clinicians on their interpretation; and integration into quality assurance processes. In resource-limited settings, the design of durable, lightweight, and modular prosthetic designs is also an important area for investment. Finally, we recommend longitudinal studies, the development of pediatric-specific patient-reported outcome measures, and research on adaptive prosthetic technologies within low- and middle-income contexts to inform sustainable context-sensitive models of care.

## 5. Conclusions

This study assessed user-reported comfort, functionality, and satisfaction levels of pediatric lower limb amputees utilizing conventional prosthetic devices within an Eastern Cape-based multi-center public-sector cohort. The results show that although most participants reported moderate to high levels of comfort and functional ability, overall satisfaction remained centered around the neutral category, indicating that positive perceptions of prosthetic performance do not necessarily translate into high overall satisfaction.

Multiple linear regression analysis demonstrated that perceived comfort and prosthetic functionality were independently associated with overall satisfaction, collectively explaining 61% of the variability in satisfaction scores. These findings highlight the importance of optimizing socket comfort and functional performance as central components of pediatric prosthetic rehabilitation while recognizing that additional factors not measured in this study are also likely to influence children’s overall prosthetic experiences.

Children with congenital limb loss reported higher levels of prosthetic functionality satisfaction than those with trauma- or disease-related amputations, emphasizing the importance of individualized rehabilitation strategies that consider differences in etiology and developmental needs.

In resource-constrained healthcare settings, where conventional prosthetic systems remain the dominant modality, these findings underscore the importance of integrating patient-reported outcome measures into routine clinical practice to better capture user experience and guide service improvement.

Overall, this study supports a shift toward growth-responsive, comfort-centered, and biopsychosocial models of pediatric prosthetic rehabilitation, in which long-term success is defined not only by prosthetic provision but by meaningful functional use, comfort, and sustained user satisfaction.

## Figures and Tables

**Table 1:** Sociodemographic characteristics of participants (*n* = 52).

Variable		Frequency (*n*)	Percent (%)
Age group	4–5	9	17.3
	6–12	21	40.4
	13–17	22	42.3
Gender	Male	35	67.3
	Female	17	32.7

**Table 2:** Primary reason for amputation by gender (*n* = 52).

Primary Reason for Amputation	Male *n* (%)	Female *n* (%)	Total *n* (%)
Congenital	16 (45.7)	11 (64.7)	27 (51.9)
Trauma	11 (31.4)	4 (23.5)	15 (28.8)
Disease	8 (22.9)	2 (11.8)	10 (19.2)
**Total**	**35 (100.0)**	**17 (100.0)**	**52 (100.0)**

(Chi-Square Test, *p* = 0.412).

**Table 3:** Primary reason for amputation by age group (*n* = 52).

Primary Reason for Amputation	4–5 Years *n* (%)	6–12 Years *n* (%)	13–17 Years *n* (%)	Total *n* (%)
Congenital	8 (88.9)	12 (57.1)	7 (31.8)	27 (51.9)
Trauma	1 (11.1)	4 (19.0)	10 (45.5)	15 (28.8)
Disease	0 (0.0)	5 (23.8)	5 (22.7)	10 (19.2)
**Total**	**9 (100.0)**	**21 (100.0)**	**22 (100.0)**	**52 (100.0)**

(Fisher’s Exact Test, *p* = 0.044).

**Table 4:** Descriptive Statistics for Comfort, Functionality, and Overall Satisfaction (*n* = 52).

Variable	Mean	SD	Minimum	Maximum
Comfort	3.54	0.87	2	5
Functionality Satisfaction	3.42	0.78	2	5
Overall Satisfaction	2.73	0.79	1	4

**Table 5:** Comfort of the Prosthesis for Daily Wear.

Comfort Rating	Frequency (*n*)	Percent (%)	Valid Percent (%)	Cumulative Percent
1 (Very uncomfortable	0	0.0	0.0	0.0
2 (Uncomfortable)	14	26.9	26.5	26.9
3 (Moderate comfort)	13	25.0	25.5	51.9
4 (Comfortable)	19	36.5	36.5	88.5
5 (Very comfortable)	6	11.5	11.8	100.0
**Total (Valid)**	**52**	**100**	**100**	-

**Table 6:** Frequency Distribution of Overall Satisfaction.

Satisfaction Level	Frequency	Percentage (%)
Very dissatisfied	3	5.8%
Somewhat dissatisfied	16	30.8%
Neutral	25	48.1%
Satisfied	8	15.4%
Very satisfied	0	0.0
**Total**	**52**	**100%**

**Table 7:** Satisfaction with prosthetic functionality according to the primary reason for amputation.

Satisfaction with Prosthetic Functionality	Congenital (*n* = 27)	Trauma (*n* = 15)	Disease (*n* = 10)	Total
Very satisfied	3 (11.1)	0 (0.0)	0 (0.0)	3 (5.8)
Satisfied	12 (44.4)	2 (13.3)	0 (0.0)	14 (26.9)
Neutral	7 (25.9)	8 (53.3)	3 (30.0)	18 (34.6)
Dissatisfied	4 (14.8)	5 (33.3)	7 (70.0)	16 (30.8)
Very dissatisfied	1 (3.7)	0 (0.0)	0 (0.0)	1 (1.9)
**Total**	**27 (100)**	**15 (100)**	**10 (100)**	**52 (100)**

Fisher’s Exact *p* = 0.006.

**Table 8:** Spearman Correlations Among Comfort, Functionality, and Overall Satisfaction.

Variables	1. Comfort	2. Functionality	3. Overall Satisfaction
1. Comfort	1.00	0.63	0.69
2. Functionality	0.63	1.00	0.51
3. Overall Satisfaction	0.69	0.51	1.00

**Table 9:** Multiple Linear Regression Analysis Examining Factors Associated with Overall Satisfaction (*n* = 50).

Predictor	B	SE	Standardized β	t	95% CI	*p*-Value
Constant	3.093	0.444	—	6.959	2.199 to 3.987	<0.001
Satisfaction with prosthetic functionality	0.326	0.086	0.383	3.781	0.153 to 0.499	<0.001
Comfort of the prosthetic for daily wear	−0.415	0.079	−0.535	−5.284	−0.574 to −0.257	<0.001

## Data Availability

The datasets presented in this article are readily available, as they are part of an ongoing study. Requests to access the datasets should be directed to the authors. The original contributions presented in this study are included in the article. Further inquiries can be directed to the corresponding author.
